# Upregulation of LHPP by saRNA inhibited hepatocellular cancer cell proliferation and xenograft tumor growth

**DOI:** 10.1371/journal.pone.0299522

**Published:** 2024-05-02

**Authors:** Chuan-Qian Bi, Tao Kang, Yu-Kang Qian, Moorim Kang, Xu-Hui Zeng, Long-Cheng Li

**Affiliations:** 1 Institute of Reproductive Medicine, School of Medicine, Nantong University, Nantong, Jiangsu, China; 2 Ractigen Therapeutics, Nantong, Jiangsu, China; National University Singapore Yong Loo Lin School of Medicine, SINGAPORE

## Abstract

Hepatocellular carcinoma (HCC) is the most common primary liver cancer worldwide and no pharmacological treatment is available that can achieve complete remission of HCC. *Phospholysine phosphohistidine inorganic pyrophosphate phosphatase* (LHPP) is a recently identified HCC tumor suppressor gene which plays an important role in the development of HCC and its inactivation and reactivation has been shown to result in respectively HCC tumorigenesis and suppression. Small activating RNAs (saRNAs) have been used to achieve targeted activation of therapeutic genes for the restoration of their encoded protein through the RNAa mechanism. Here we designed and validated saRNAs that could activate LHPP expression at both the mRNA and protein levels in HCC cells. Activation of LHPP by its saRNAs led to the suppression of HCC proliferation, migration and the inhibition of Akt phosphorylation. When combined with targeted anticancer drugs (*e*.*g*., regorafenib), LHPP saRNA exhibited synergistic effect in inhibiting *in vitro* HCC proliferation and *in vivo* antitumor growth in a xenograft HCC model. Findings from this study provides further evidence for a tumor suppressor role of LHPP and potential therapeutic value of restoring the expression of LHPP by saRNA for the treatment of HCC.

## Introduction

Hepatocellular carcinoma (HCC) is the most common primary liver cancer and the third leading cause of cancer-related deaths worldwide. No pharmacological treatment is available that can achieve complete remission of HCC. Depending on tumor staging, liver function, and underlying physical status of patient, more effective local and systemic therapies may be administered [1]. Surgical resection is the mainstay of liver cancer treatment, and liver transplantation and embolization are all available clinical options [2]. However, due to the strong compensatory function of liver, the onset of liver tumors is usually more insidious than that of other diseases. Most of the patients with advanced hepatocellular carcinoma have already missed the best time for radical surgery when they are diagnosed. Moreover, conventional chemotherapy cannot improve the quality of life and survival rate of them, so transcatheter arterial chemoembolization (TACE) and transcatheter arterial chemotherapy infusion (TACI) have become the most common palliative treatments [3]. Patients with advanced hepatocellular carcinoma have more treatment options now. Due to the unique metabolic and immunosuppressive environment of liver cancer [4], molecularly targeted therapies for liver cancer, such as molecular targeted therapies and immunotherapy [5, 6], have undergone significant changes and have also become an important therapeutic avenue. Sorafenib, a multikinase inhibitor, is a recommended targeted agent for the effective treatment of advanced hepatocellular carcinoma with good tolerability, safety, and significant clinical value [7]. More recently, lenvatinib, regorafenib, cabozantinib and ramucirumab have emerged as new treatment options [8]. A recent study has shown that brusatol (BT) inhibits HCC metastasis by downregulating epithelial-mesenchymal transition (EMT) [9]. Tris(dibenzylideneacetone)dipalladium(0) (Tris DBA) inhibits tumor progression in HCC and multiple myeloma (MM) preclinical models by regulating the STAT3 signaling pathway [10]. Recent genomic profiling efforts have provided deep understanding of molecular pathogenesis of HCC and a number of overactivated tumor driver pathways have been identified such as the Wnt and mTOR signaling pathways and but only a few are druggable targets by conventional treatment [11–13].

*Phospholysine phosphohistidine inorganic pyrophosphate phosphatase* (LHPP) is a recently identified tumor suppressor gene, which plays a key role in the development of HCC. LHPP is downregulated in both human and mouse HCC, and its inactivation and reactivation lead to hepatocellular carcinogenesis and inhibition, respectively [14]. Subsequent studies have confirmed that LHPP expression level is negatively correlated with cell cycle progression and metastasis in human HCC tissues and forced overexpression of LHPP inhibited the growth and metastasis of various human hepatoma cells [15]. A similar tumor suppressor role has been reported in other types of cancer such as cervical cancer [16], gastric cancer [17, 18], thyroid cancer [19], bladder cancer [20], colorectal cancer [21] and glioblastoma [22]. Therefore, restoring the function of LHPP may serve as a potential therapeutic target for HCC and other types of tumors.

RNA activation (RNAa) is a biological mechanism by which promoter-targeted duplex RNAs can induce target gene expression in a sequence-specific manner and such duplex RNAs are known as small activating RNA (saRNA) [23]. saRNA has been shown to be able to activate a variety of genes including tumor suppressor genes [24]. One such saRNA, MTL-CEBPA, that targets and activates the tumor suppressor gene CEPBA, is being developed as a therapeutic for advanced HCC and has entered phase II trial [25–27].

**Fig 1:**
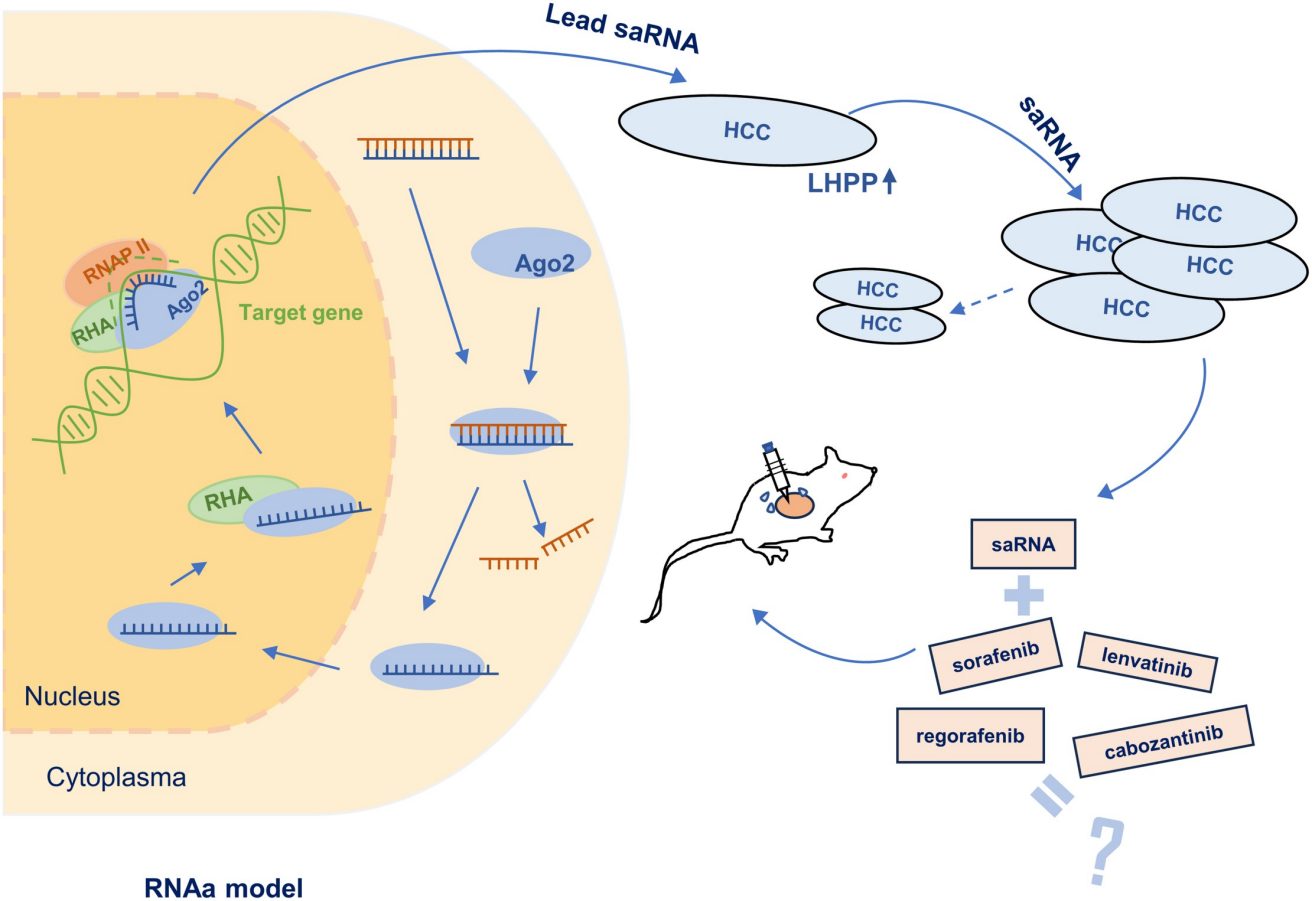
Schematic of LHPP saRNA for treating HCC. Promoter-targeted saRNA joins with Ago2, forming an active complex after strand separation. In the nucleus, Ago2 may engage RNA helicase A (RHA) to unwind DNA, allowing the RNA guide strand to bind the target DNA. saRNAs were designed to target the promoter of LHPP gene to increase its expression in HCC cells, resulting in inhibition of cell proliferation and migration. Combined with targeted anticancer drugs, these saRNAs exert a synergistic effect in reducing in vitro HCC proliferation and in vivo tumor growth in a xenograft HCC model.

We hypothesize that saRNA can be utilized to activate LHPP and thereby inhibit the growth of HCC. In the present study, we designed and validated saRNAs which robustly induced LHPP mRNA and protein expression. One of the lead saRNAs, RAG7-133, showed activity in inhibiting HCC proliferation and migration and a significant synergistic effect with targeted anticancer drugs in suppressing *in vitro* tumor cell proliferation and *in vivo* tumor growth. It is concluded that targeted activation of LHPP by saRNA may provide a novel therapeutic strategy for HCC (Fig 1).

## Materials and methods

### saRNA design

A 1-kb LHPP promoter sequence upstream of its transcription start site (TSS) was retrieved from UCSC genome database excluding an Alu repeat region (-647 bp to -198 bp) and used as a template for selecting saRNA targets. Target sequences with the following features were excluded including a GC content less than 35% or greater than 65%, 5 or more consecutive nucleotides, and 3 or more di- or tri-nucleotide repeats, resulting 290 remained target sequences based on which duplex RNAs of 21 nt were designed and chemically synthesized.

### Chemicals

Sorafenib (Cat#S1040), lenvatinib (Cat#S1164), regorafenib (Cat#S1178) and cabozantinib (Cat#S1119) were obtained from Selleck (Houston, TX, USA).

### Cell culture and treatment

Human HCC cell lines Huh7 and HepG2 were obtained from Nanjing Cobioer Biosciences (Nanjing, China). Both cells were cultured in Dulbecco’s modified Eagle’s medium (Cat#11995065, Gibco, Grand Island, NY, USA) supplemented with 10% fetal bovine serum (Cat#10099141C, Gibco, Australia) and 1% Penicillin Streptomycin (Cat#15140122, Gibco), cultured at 37°C in a 5% CO_2_ environment.

Duplex RNAs were transfected into cells using Lipofectamine^™^ RNAiMAX (Cat#13778075, Invitrogen, USA) by following the reverse transfection method provided in the manufacturer’s manual for 72 hours unless otherwise stated.

For combinatory treatment with chemical compounds, HepG2 cells were plated in 96-well plates and reverse transfected with duplex RNAs for 24 hours, and then compounds (ssorafenib 1 μM, lenvatinib 5 μM, regorafenib 1 μM, or cabozantinib 5 μM) or DMSO control (0.1%) was added directly to the medium and the cells were further cultured for 48 hours before analysis.

### Reverse transcription real-time quantitative polymerase chain reaction (RT–qPCR)

Total RNA was isolated from treated cells using the MagBind Particles (Cat#MB-10, Magen, China) in accordance with the manufacturer’s instructions. One microgram of the total RNA was reverse transcribed into complementary DNA (cDNA) using the PrimeScript^™^ RT reagent Kit (Cat#RR047A, Takara, Japan). The resulted cDNA was subjected to qPCR using TB Green^®^ Premix Ex Taq^™^ II (Tli RNaseH Plus) (Cat#RR820, Takara). The reaction conditions were as follows: 1 cycle of pre-incubation at 95°C for 30 sec; 40 cycles of amplification at 95°C for 5 sec and 60°C for 30 sec; 1 cycle of melting curve at 95°C for 5 sec and 65°C for 1 min; and 1 cycle of cooling to 40°C for 30 sec. The HPRT1 and TBP genes were also amplified and their geometric means of amplification was used to normalize that of LHPP. In addition, RT–PCR of the obtained cDNA was performed using One Step TB Green^®^ PrimeScript^™^ RT-PCR Kit II (Perfect Real Time) (Cat#RR086A, Takara). The reaction conditions were as follows: 1 cycle of pre-incubation at 42°C for 5 min and 95°C for 10 sec; 40 cycles of amplification at 95°C for 5 sec, 60°C for 20 sec and 72°C for 10 sec; 1 cycle of melting curve at 95°C for 5 sec and 65°C for 15 sec; and 1 cycle of cooling to 40°C for 30 sec. The HPRT1 and TBP genes were also amplified to serve as reference genes. The primers used for RT-qPCR and one step RT-PCR are listed in S1 Table. Each experiment was repeated at least 3 times unless otherwise specified.

### Western blotting assay

Cells were lysed with RIPA buffer (Cat#P0013B, Beyotime, China) supplemented with 1% Protease Inhibitor Cocktail (Cat#P8340, Sigma, USA) and 1% Phenylmethanesulfonyl fluoride solution (Cat#93482, Sigma) on 4°C for half an hour, and proteins were collected from the lysed cells after centrifugation at 12000 rpm, 4°C, 15 min. Protein concentration was determined using BCA Protein Assay Kit (Cat#P0011, Beyotime). The protein samples were then separated by electrophoresis on a 10% sodium dodecyl sulfate (SDS)-polyacrylamide gel and electroblotted onto PVDF Membrane (Cat#IPVH00010, Millipore, USA). The membranes were blocked with blocking solution (5% nonfat dry milk) for 2 hours at room temperature, incubated with anti-LHPP polyclonal antibody (Cat#PA5-89768, Invitrogen; 1:1000 dilution), anti-Akt (Cat#4691, Cell Signaling Tech, USA; 1:1000 dilution), anti-Phospho-Akt (Ser473) (Cat#4060, Cell Signaling Tech; 1:2000 dilution), or anti-α/β-tubulin antibody (Cat#2148s, Cell Signaling Tech; 1:5000 dilution) as the primary antibodies at 4°C overnight. Secondary anti-rabbit antibody (Cat#7074s, Cell Signaling Tech; 1:10,000 dilution) was then incubated at room temperature for 1 hour. The membranes were imaged and recorded using a multifunctional gel imaging system (ChemiDocTM MP, Bio-Rad, USA).

### CCK-8 cell proliferation assay

Cells were seeded in 96-well plate and reverse transfected for 72 hours. At the end of transfection, culture medium was removed from treated cells. 100 μL fresh medium and 10 μL CCK-8 (Cat#CK04, Dojindo, Japan) solution were added to each well and incubated at 37°C for 1 hour. A microplate reader (TECAN, Switzerland) was used to measure the OD value of each experimental well at 450 nm.

### CellTiter-Glo luminescent cell viability assay

Cells were seeded in 96-well plate and reverse transfected for 72 hours. At the end of transfection, 100 μl of CellTiter-Glo Reagent (Cat#G7570, Promega, USA) was added to each well and mixed for 30 min on an orbital shaker to induce cell lysis. The plates were left to incubate at room temperature for 10 minutes to stabilize the luminescent signal and then measured in a microplate reader.

### Wound healing assay

Cells were plated in 24-well plates for 24 hours until a confluent layer was formed. Scratches were then introduced to the confluent cell layer by scratching across the cell layer using the tip of a sterile 200 μL pipette. Culture medium was removed, and cells were washed with PBS to remove cell debris. The cells were then transfected with duplex RNA using RNAiMAX and incubated for another 72 hours. Cell images were taken at 0-, 24-, 48- and 72-hours post-transfection. Wound closure was measured and calculated using the Image J software (National Institutes of Health, NIH, USA).

### Formulation of saRNA for *in vivo* use

saRNA was formulated using in vivo jetPEI (Cat#201-10G, Polyplus-transfection, France) by following the instruction of the manufacturer. Briefly, saRNA was diluted in 10% glucose solution to obtain Solution A. in vivo-jetPEI was diluted in 10% glucose solution to obtain Solution B. Equal volume of Solution A and Solution B was then mixed and let to stand at room temperature for 15 minutes before use. The resulted final saRNA formulation had a nitrogen to phosphorus ratio of 8 and a final glucose concentration of 5%.

### Animals

BALB/c nude athymic mice were obtained from GemPharmatech LLC (Nanjing, China). All mice were housed under temperature and humidity-controlled conditions. HepG2 cells (5×10^6^) were subcutaneously injected into the right flank of the mice. After 25 days when tumor volume reached 200 mm^3^, the animals were randomly divided into 4 groups (Vehicle control, RAG7-133, Regorafenib, RAG7-133 + Regorafenib) with 6 mice in each group. Treatments were given on the 1st, 4th, 7th, and 10th day. RAG7-133 formulated in in vivo-jetPEI was intratumorally injected at the dose of 1 mg/kg for RAG7-133. Regorafenib was administered by gavage at the dose of 3 mg/kg daily on day 1 to day 12. Tumor long diameter and short diameter were measured using a vernier caliper every other day after the first dose and tumor volume (V) was calculated using the formula V = (L×W^2^)/2 where L is the longest diameter and W the diameter parallel to the surface of the tumor mass and perpendicular to the long diameter. Respecting the animal ethics rules, the mice were fasted overnight and then euthanised by inhalation of carbon dioxide. The animal procedure was approved by Institutional Animal Care and Use Committee of Ractigen Therapeutics.

### Synergistic effect analysis

To determine whether combinatory treatments elicited an synergistic effect on cell proliferation, combination index (CI) was calculated using Compusyn© version 1.0 software (ComboSyn, Inc., Paramus, NJ, USA) following the method of Chou [28, 29]. A CI value <1, = 1, and >1 indicate synergism, additive effect, and antagonism, respectively. And Combenefit software was also used to perform synergism determination, which was analyzed using classical models, including the Loewe, the Bliss and the Highest Single Agent (HSA) models [30].

### Statistical analysis

Data was presented as means ± standard deviations (SD) of at least two independent experiments. For quantitative data, analysis of variance (ANOVA) was used to assess statistical significance followed by Dunnett’s multiple comparisons test. Differences were deemed significant when the *p* value was less than 0.05. GraphPad Prism software (GraphPad Software, San Diego, CA, USA) was used for the analyses.

## Results

### Screen and identification of LHPP saRNAs

**Fig 2:**
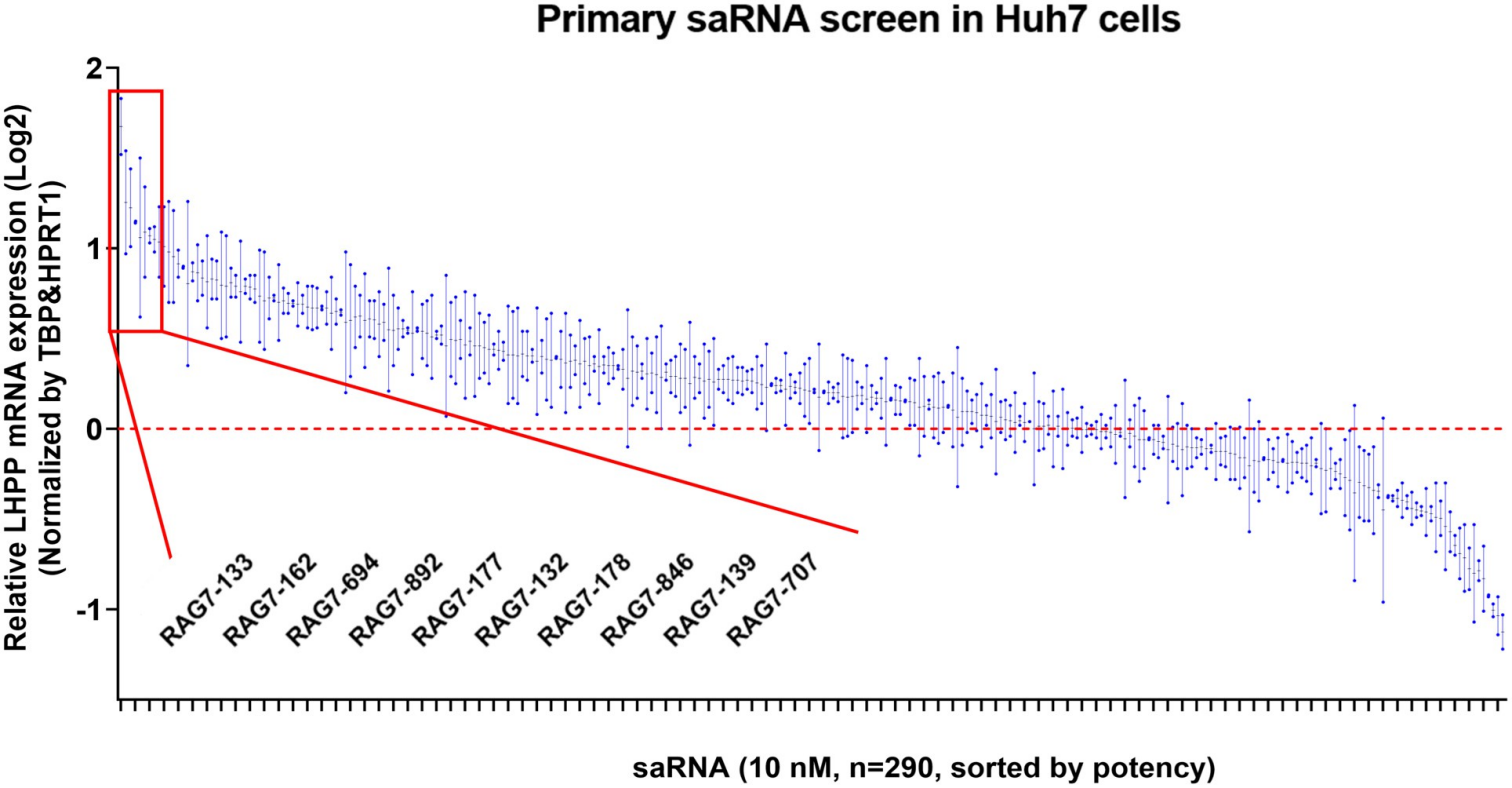
saRNA design and screen. 290 duplex saRNA candidates targeting the LHPP gene promoter sequence were designed, synthesized and individually transfected into Huh7 cells for 72 hours. The transfected cells were analyzed for LHPP mRNA expression by one-step RT-qPCR. HPRT1 and TBP were also analyzed as internal reference genes and their geometric means was used to normalize the expression of LHPP. The data represents the mean ± SD of two replicate transfection wells and is plotted as log_2_ values on the y axis. 290 saRNA candidates are not shown due to the limited capacity of the x-axis. The name of top 10 lead saRNAs are shown.

To identify saRNAs that can activate LHPP expression, 290 dsRNAs were designed to target the LHPP promoter and individually transfected into Huh7 cells. A dsRNA that does not target any known gene sequence, dsCon2, was also synthesized and served as a control dsRNA. saRNAs were transfected using RNAiMAX transfection reagent at a concentration of 10 nM in serum-free conditions into Huh7 cells. LHPP mRNA expression was detected using one-step RT-qPCR at 72 hours after transfection. The resulted screen data is summarized in Table 1 and plotted in Fig 2. Compared to mock transfection, 202 (~69.7%) of the 290 saRNAs transfected showed an activating effect and 88 (30.3%) exhibited an inhibiting effect on the expression of LHPP mRNA. This result indicates that most (~ one-third) of the LHPP promoter-targeting dsRNAs had an activating effect on LHPP expression.

**Table 1:** saRNA screen results.

Category	No. of dsRNAs (%)
Activating dsRNAs (>1 fold)	202 (69.7%)
≥ 1.5 fold	57 (19.7%)
<1.5 fold ~ ≥ 1.2 fold	77 (26.6%)
Inhibitory dsRNAs (<1 fold)	88 (30.3%)
Total	290 (100%)

### Validation of lead saRNAs in Huh7 and HepG2 cells

**Fig 3:**
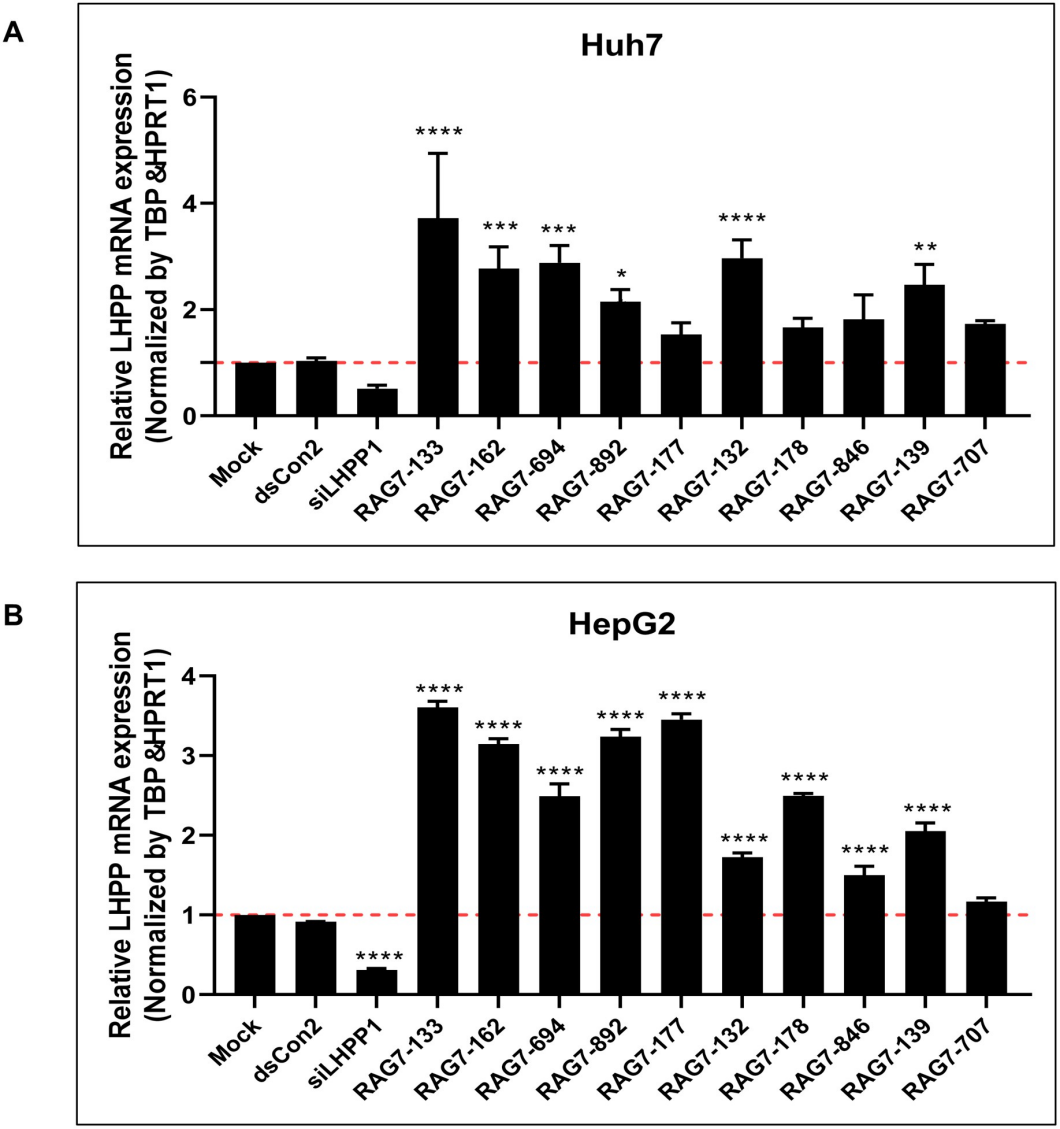
Lead saRNA induced LHPP mRNA expression in Huh7 and HepG2 cells. Lead saRNAs at a concentration of 10 nM were transfected into Huh7 cells (A) and HepG2 cells (B) by using RNAiMAX for 72 h. LHPP mRNA expression was detected by RT-qPCR. HPRT1 and TBP genes were also amplified to serve as internal control for RNA loading. Data is plotted as fold changes relative to mock transfection and represents the mean ± SD of two independent experiments. *P<0.05, ** *P*<0.01, *** *P*<0.001, **** *P*<0.0001.

To confirm the induction of LHPP mRNA expression by lead saRNAs in Huh7 and HepG2 cells, 10 saRNAs (RAG7-133, RAG7-162, RAG7-694, RAG7-892, RAG7-177, RAG7-132, RAG7-178, RAG7-846, RAG7-139, RAG7-707) were selected and transfected into Huh7 cells (Fig 3A) and HepG2 cells (Fig 3B) at a concentration of 10 nM for 72 hours. LHPP mRNA expression was evaluated by RT-qPCR. As shown in Fig 3A and 3B, all saRNAs induced LHPP mRNA expression in both cell lines ranging from 1.3 to 5.1 fold in Huh7 cells and 1.1 to 3.7 fold in HepG2 cells. RAG7-133 was the most potent saRNA consistently inducing LHPP expression in both cell lines and was thus selected for more focused analysis.

### Lead saRNA induced LHPP protein expression and suppressed phosphorylated Akt

**Fig 4:**
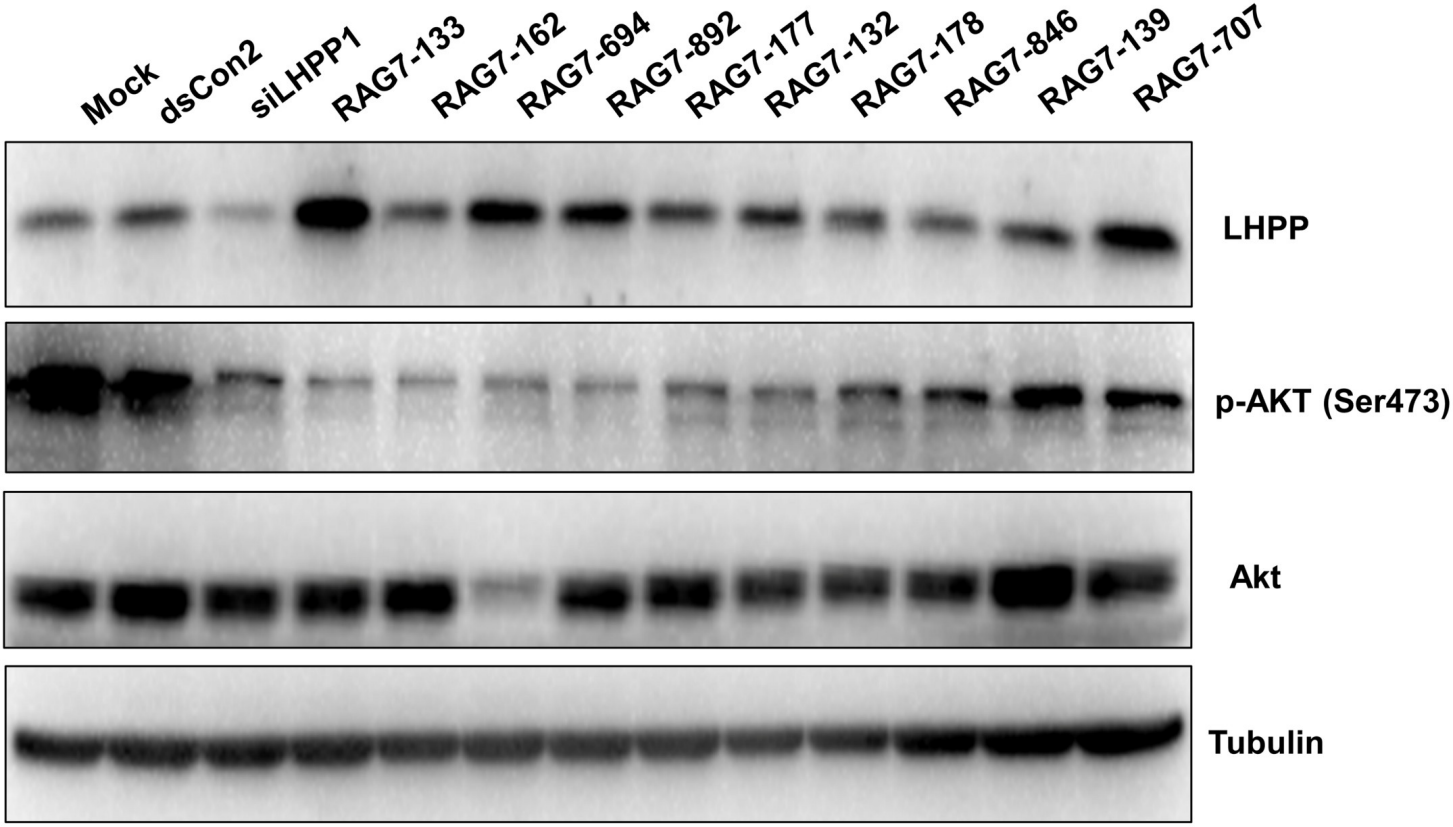
Lead saRNAs upregulated protein expression of LHPP and inhibited Akt phosphorylation. Lead saRNAs at a concentration of 10 nM were transfected into Huh7 cells by using RNAiMAX for 72 h. Protein levels of LHPP, pAkt (Ser473) and Akt was detected by western blotting assay. Tubulin was also blotted as an internal reference for protein loading.

To further confirmed LHPP activation by lead saRNAs, Huh7 cells were transfected with lead saRNAs (RAG7-133, RAG7-162, RAG7-694, RAG7-892, RAG7-177, RAG7-132, RAG7-178, RAG7-846, RAG7-139, RAG7-707) as well as a LHPP siRNA for 72 hours. Protein samples from the transfected cells were subject to western blotting analysis to detect the protein levels of LHPP, Akt and Phospho-Akt (Ser473). As shown in Fig 4, all saRNAs induced LHPP protein expression to varying degrees with RAG7-133 being the strongest inducer. All lead saRNAs did not cause an obvious change to the protein level of total Akt protein except RAG7-694 which caused a significant decrease of Akt protein, possibly through an off-targeting effect. Interestingly, pAkt (Ser473) levels was decreased by most of the duplex RNA transfection including LHPP siRNA (siLHPP1).

### RAG7-133 inhibited HCC proliferation and migration

**Fig 5:**
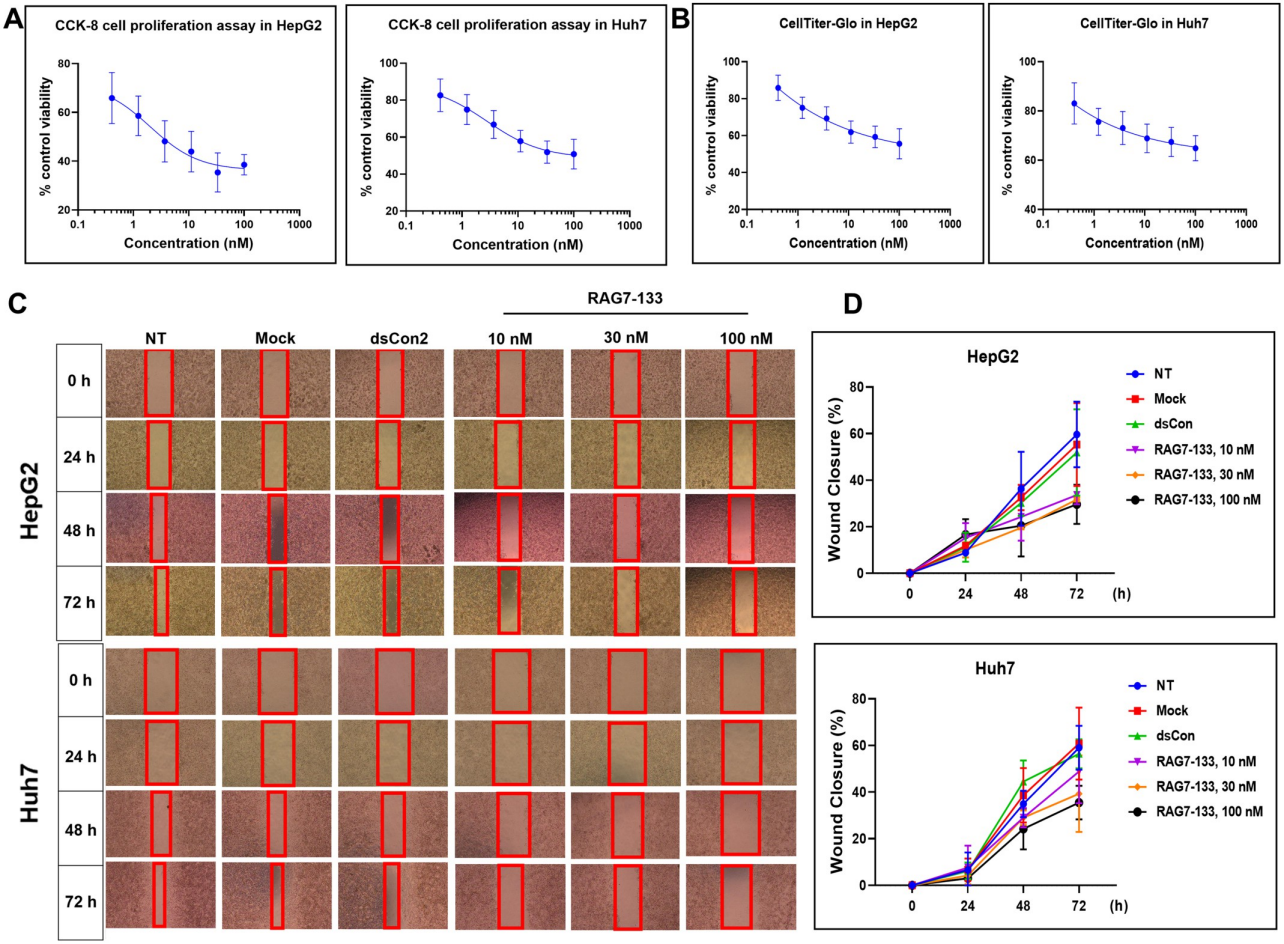
Effects of RAG7-133 on HCC cell proliferation and migration. HepG2 and Huh7 cells were transfected with RAG7-133 at the indicated concentrations for 72h (for cell proliferation assay) or for the indicated period (for wound healing assay). The transfected cells were analyzed for cell proliferation by CCK-8 and CellTiter-Glo assays and wound healing assay. (A) Effect of RAG7-133 on cell viability detected by CCK-8 cell proliferation assay in HepG2 and Huh7 cell lines. (B) Effect of RAG7-133 on cell viability detected by CellTiter-Glo assay in HepG2 and Huh7 cell lines. (C) Wound healing assay images of different treatment groups were obtained after transfection at 0-, 24-, 48- and 72-hours in HepG2 and Huh7 cell lines. (D) Quantified cell migration rate for the images (C), expressed as a percentage of area reduction or closure of wound healing assay in HepG2 and Huh7 cell lines.

We chose the most potent saRNA RAG7-133 for further functional studies. RAG7-133 which has a target on the LHPP promoter between –133 and –152 position relative to LHPP TSS (S1 Table) was transfected into HepG2 and Huh7 at concentrations ranging from 0.41 nM to 100 nM for 72 hours and cell proliferation was assessed by CellTiter-Glo luminescent cell viability assay and CCK-8 cell proliferation assay. As shown in Fig 5A and 5B, a dose-dependent inhibition of proliferation was observed in both cell lines with a maximum inhibition of 36.3% and 51.8% by RAG7-133 at 100 nM with a calculated IC_50_ at 3.15 nM and 0.68 nM in HepG2 and Huh7 cells respectively.

Further, a wound healing assay was performed to determine whether RAG7-133 could influence cell motility and migration. RAG7-133 was transfected at 10, 30 and 100 nM into HepG2 and Huh7 cells. The transfected cells were wounded at 24 hours and cell healing images were taken at 24, 48 and 72 hours (Fig 5C). As shown in Fig 5C and 5D, RAG7-133 transfected cells exhibited a significant slowdown in the healing rate and a significant decrease in the area of closure compared to the control groups (Mock and dsCon2).

### Synergistic activity of RAG7-133 with targeted antitumor drugs in inhibiting proliferation of HepG2 cells

**Fig 6:**
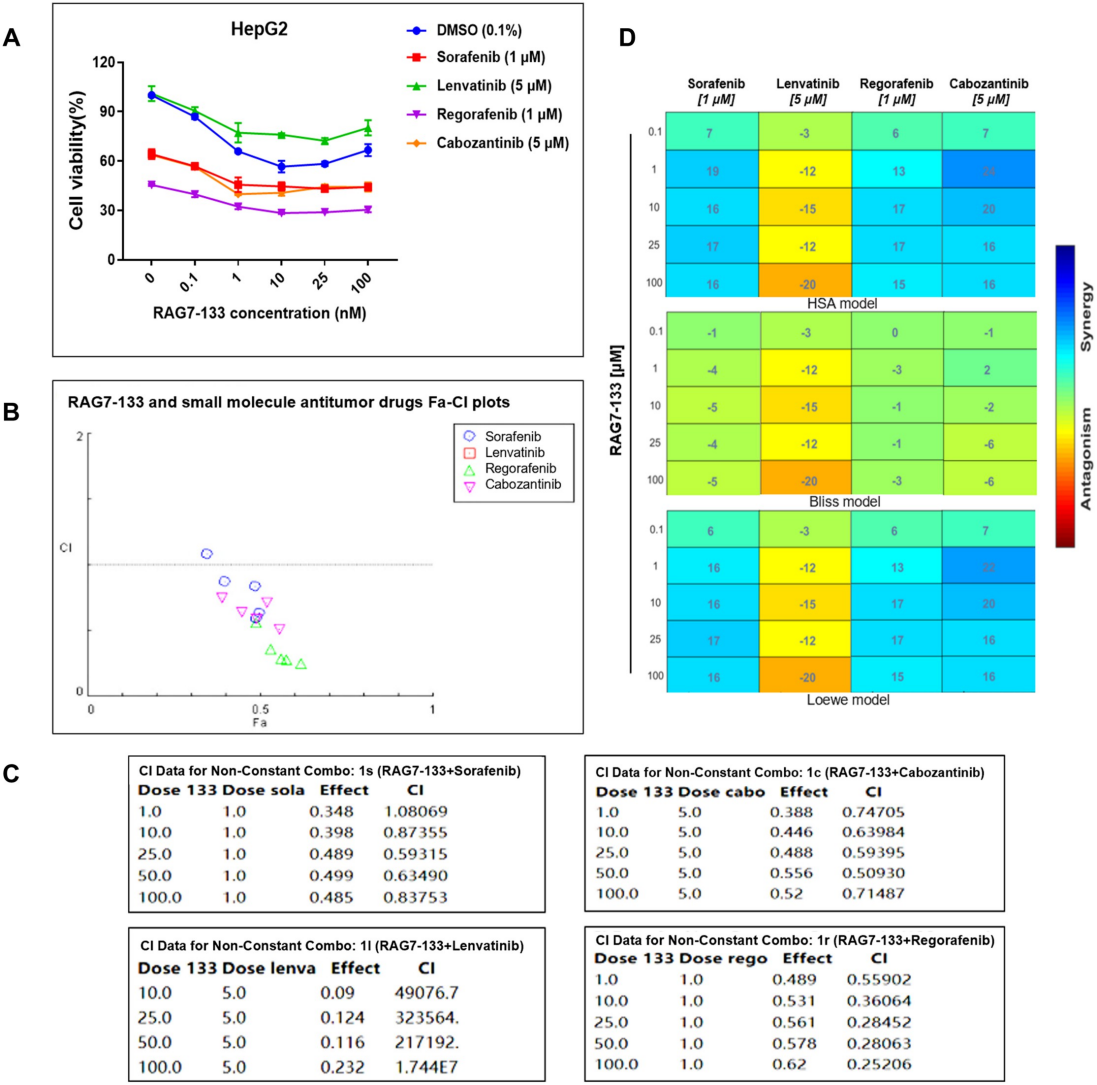
Synergistic effect of RAG7-133 with small molecule antitumor drugs in inhibiting HCC proliferation. (A) HepG2 cells were transfected with the indicated concentrations of RAG7-133 and co-treated with the indicated small molecule antitumor drugs. CCK-8 cell proliferation assay was used to evaluate cell viability. The cell viability of the saRNA treated group was expressed as a percentage relative to the mock transfected group. (B) Fa-CI plots (Fraction affected combination index). Compusyn© version 1.0 software was used to draw the combination index diagram by using combination index (CI). Fa-CI plot shows CI on y-axis as an effect level function of the drug combination on x-axis (Fa: fraction of dead cells compared to control). Data points below the horizontal line (CI = 1) indicate synergy. Lenvatinib was not shown in the graph due to antagonism. (C) CI data for non-constant combo. (D) Synergy distribution, obtained by Combenefit software with the Loewe, the Bliss and the Highest Single Agent (HSA) models.

To explore potential synergistic activity of RAG7-133 with approved targeted antitumor drugs for HCC including sorafenib, lenvatinib, regorafenib and cabozantinib in inhibiting HCC proliferation, RAG7-133 was transfected into HepG2 at different concentrations in the absence or presence of the small molecular drugs for 72 hours. The treated cells were subject to CCK-8 cell proliferation assay. As shown in Fig 6A, in the presence of sorafenib, regorafenib or cabozantinib, more profound inhibition of cell proliferation was induced compared to the DMSO vehicle control. In the presence of each of the small molecular drugs except Lenvatinib, saRNA transfection caused an enhanced antiproliferative effect compared to mock transfection (saRNA concentration at 0 nM). As shown in Fig 6B and 6C, Calculation of the combination index (CI) showed that RAG7-133 at 25, 50, 100 nM achieved strong synergy (0.1 < CI < 0.3) with regorafenib. RAG7-133 at 1, 10 nM with regorafenib, RAG7-133 at 25, 50 nM with sorafenib, RAG7-133 at 10, 25, 50 nM with cabozantinib also achieved synergy (0.3 < CI < 0.7). RAG7-133 at 100 nM with Sorafenib, RAG7-133 at 1, 100 nM with cabozantinib achieved moderate synergy (0.7 < CI < 0.85). RAG7-133 at 10 nM with Sorafenib achieved slight synergy (0.85 < CI < 0.9). The Combenefit software was used to obtain synergy distributions using Loewe, Bliss, and Highest Single Agent (HSA) models. Lenvatinib has obvious antagonism effect. Interestingly, RAG7-133 at 1 μM showed high synergy with cabozantinb in both the HSA model and the Loewe model (Fig 6D).

### RAG7-133 inhibited HepG2 xenograft tumor growth

**Fig 7:**
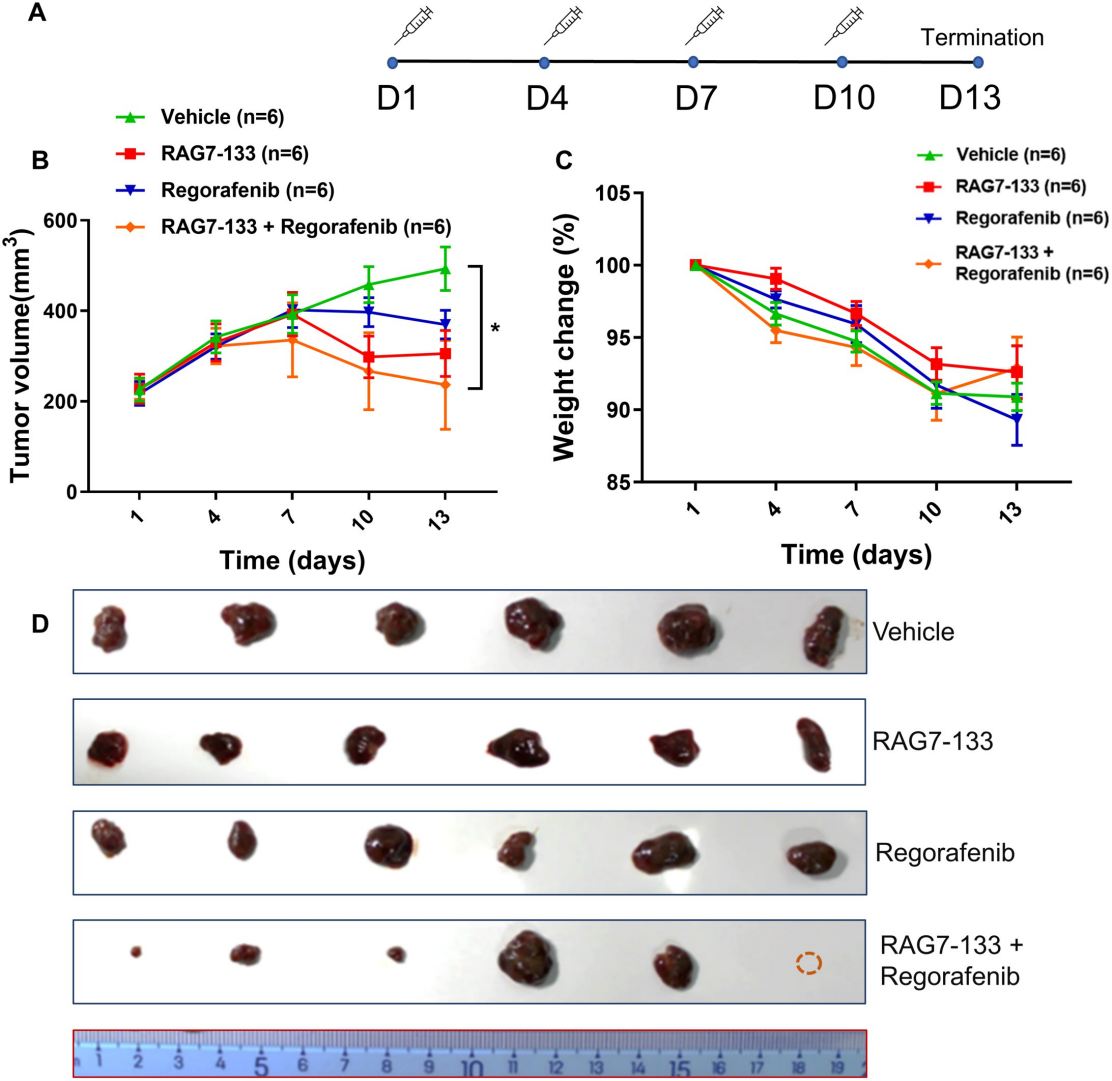
Antitumor effect of RAG7-133 and its synergistic activity with targeted antitumor drugs in HepG2 xenograft tumor model in mice. (A) Schematic of experimental timeline. (B) Measurements of tumor volume at different time points. (C) Body weight changes in different groups. (D) Image of harvested tumor in different groups. *P<0.05.

To further assess *in vivo* antitumor activity of RAG7-133 alone and in combination with the targeted therapy drug Regorafenib, xenograft tumor models were created in nude mice by subcutaneously inoculating 5 × 10^6^ HepG2 cells. After 25 days, tumor bearing mice were randomly divided into 4 groups (n = 6): Vehicle control, RAG7-133, Regorafenib, and combination of RAG7-133 and Regorafenib. RAG7-133 formulated in *in vivo*-jetPEI was administrated by intratumoral injections on the 1st, 4th, 7th, and 10th day (Fig 7A). Vehicle control (DMSO) and regorafenib were administrated daily by gavage. The study was terminated 13 days after the first dose.

As shown in Fig 7B, starting from the third dose, tumors treated with RAG7-133 alone, regorafenib and their combination started to regress compared to tumors treated with vehicle control. More profound tumor regression was observed in RAG7-133 treated tumors than Regorafenib treated tumor with the greatest regression occurred in the combo treatment group. Tumor volume increased by only 4% in the combo treatment group and increased by 34% in the RAG-133 group, while the tumor volume in the Regorafenib group alone increased by 70% on day 13 (*P* < 0.05). The body weight data showed that weight in the combo treatment group decreased more significantly on the 4th, 7th, and 10th day compared to the other groups but increased to a level consistent with the other groups on day 13 (Fig 7C). Tumor shrinkage from the treatments can also be appreciated from the harvested tumors at the end of the study. Significantly smaller tumors occurred in the combo treatment group with one disappeared tumor resulting 5 visible tumors only (Fig 7D).

## Discussion

Early detection, early diagnosis and early treatment are crucial to the prognosis of tumor diseases. But due to the strong compensatory function of the liver, hepatocellular carcinoma has an insidious onset, and it is often at an advanced stage when patients develop obvious clinical symptoms. As one of the common causes of cancer-related deaths globally, hepatocellular carcinoma has a huge gap in unmatched systemic treatments, and therapeutic options are in dire need of a breakthrough [31]. LHPP is a tumor suppressor gene that can play an important role in the development of hepatocellular carcinoma discovered recently. And for this gene, no targeted drug has been introduced yet. Inactivation and reactivation of LHPP has been shown to result in respectively HCC tumorigenesis and suppression, and clinical data show that median survival is reduced by nearly 2 years in patients with low expression of LHPP. Reintroducing the genetic information of LHPP and increasing its expression level can effectively inhibit the growth and metastasis of liver tumors [15], and significantly reduce the tumor load and liver function impairment [14]. LHPP has the potential to be a tumor biomarker and therapeutic target for hepatocellular tumors. RNAa, as an emerging technology for regulating gene expression, has great potential for the treatment of malignant tumors. It can specifically and efficiently target genes [32] and regulate their expression with long-lasting and persistent effects. In this way, utilizing known RNAa techniques to target LHPP may be a solution for hepatocellular carcinoma. In this study, we designed and identified potent saRNAs that activated LHPP expression in HCC cells and caused inhibition of cell proliferation and tumor growth in a xenograft model. Combination of lead saRNA RAG7-133 with targeted antitumor drugs exhibited substantial synergistic activity in inhibiting HCC proliferation *in vitro* and tumor growth *in vivo*.

saRNAs are used to achieve targeted activation of specific therapeutic genes while also restoring the expression of their encoded proteins. Lead saRNA upregulates LHPP mRNA along with its protein expression levels. Almost half of HCC patients show the presence of aberrant PI3K-AKT-mTOR signaling [33], including loss of the tumor suppressors PTEN, TSC1, and TSC23. Previous studies have shown a negative correlation between p-AKT (Ser473) and LHPP protein expression levels in liver tumors of PTEN and TSC1 double knockout mice [14]. Western blot showed that saRNAs up-regulate LHPP protein expression while causing down-regulation of AKT (Ser473) phosphorylation levels [34]. None of the lead saRNAs caused significant changes in total AKT protein levels except for RAG7-694 which caused a significant reduction potentially due to off-target effects. RAG7-133, which had the best effect, was selected for subsequent experiments. CCK-8 cell proliferation assay was associated with changes in cellular mitochondrial respiratory activity, and CellTiter-Glo was detected based on the presence of ATP as well as the degree of cellular metabolic activity [35]. Wound healing assay were performed to find that it inhibited the proliferation and migration of hepatocellular carcinoma cells in a dose-dependent manner. Clinical experience shows that the combination of multiple drugs is more effective than single drug therapy [36]. In the past few years, small molecule antitumor drugs such as the multikinase inhibitors lenvatinib, regorafenib and cabozantinib been approved for the treatment of HCC but with limited clinical efficacy [11]. Here, we use the RAG7-133 in combination with the first-line agents for HCC sorafenib or lenvatinib, and the second-line targeted agents regorafenib or cabozantinib separately [37]. In HepG2 cells, the combination of Sorafenib, regorafenib and cabozantinib with RAG7-133 showed excellent tumor cell inhibition especially at higher RAG7-133 concentrations. This confirms the possibility of saRNA in combination with targeted therapeutic agents for tumor treatment. Some of the tumor volume trends were not as expected, which was presumably due to individualization of the model mice. In fact, in order to reduce the impact of different risk factors and microenvironmental differences, the treatment of patients with liver cancer is gradually moving towards more appropriate diagnosis and treatment methods and individualized precision treatment [38]. It may also be due to the lack of a suitable in vivo delivery system for saRNA, which is still a technical challenge for nucleic acid-based drugs [39]. In the currently approved oligonucleotide therapeutics, the main delivery modes are ligand coupling and lipid nanoparticles (LNPs) [40]. But due to their special self-acting and physicochemical properties, it is difficult to achieve satisfactory pharmacokinetic and pharmacodynamic results with pure chemical modification [41], but may be a factor in increasing efficient action and target specificity. In this paper, we focus on validating the inhibition of hepatocellular carcinoma cell proliferation and xenograft tumor growth, which is more intuitively reflected by the effect of action. Targeted gene activation by saRNA is being actively pursued as a therapy for a variety of diseases including cancer. Current cancer treatments heavily depend on approaches that suppress key oncogenic signaling pathways. Cancer stems from an imbalance of over-activated oncogenes and inactivated tumor suppressor genes. Approaches that can restore inactivated tumor suppressor genes is equally important in developing anticancer drugs. In this regard, MTL-CEBPA, a saRNA drug that targets and activates CEPBA, is being developed as a therapeutic for advanced HCC and has entered phase II trial [26].

LHPP is a tumor suppressor gene originally identified in HCC and later in many other tumor types [14]. In the present study, we designed and validated saRNAs that efficiently upregulated LHPP mRNA expression in HCC cell lines. One of the lead saRNAs, RAG7-133, can significantly upregulate the expression of LHPP mRNA and protein and thereby inhibit HCC proliferation and migration and has synergy with a number of small molecular anticancer drugs in inhibiting HCC proliferation. This synergistic effect could be validated in xenograft HCC models in which combo treatment of tumors with RAG7-133 and Regorafenib resulted in significant tumor regression than each of them used alone. This study may provide a novel therapeutic approach for HCC by targeted activation of tumor suppressor gene LHPP.

## Supporting information

S1 Table Sequences of PCR primers and lead saRNA RAG7-133. (DOCX)

S1 Raw images (PDF)
