# Supplementary material for: Upregulation of LHPP by saRNA inhibited hepatocellular cancer cell proliferation and xenograft tumor growth
Source: PLoS One. 2024 May 2;19(5):e0299522. doi: 10.1371/journal.pone.0299522 (PMC11065268; doi:10.1371/journal.pone.0299522)
Supplement: S1 Table — (DOCX) [file pone.0299522.s001.docx]

**S1 Table. Sequences of PCR primers, dsCon2, siRNA and lead saRNAs**

| **PCR primers** | | | | | |
| --- | --- | --- | --- | --- | --- |
| **Gene** | **Forward** | | **Reverse** | | **Product size (bp)** |
| **TBP** | ATAATCCCAAGCGGTTTGCT | | CTGCCAGTCTGGACTGTTCT | | 126 |
| **HPRT1** | ATGGACAGGACTGAACGTCTT | | TCCAGCAGGTCAGCAAAGAA | | 113 |
| **LHPP** | AAGGCGCTTGAGTATGCCTG | | GTGGGCTTCCACTCCTATCG | | 102 |
| **dsCon2, siRNA and lead saRNAs** | | | | | |
| **Name** | | **Sense** | | **Antisense** | |
| **dsCon2** | | ACUACUGAGUGACAGUAGA[dT][dT] | | UCUACUGUCACUCAGUAGU[dT][dT] | |
| **siLHPP1** | | GAAGUUCAGAGCCGCUCAA[dT][dT] | | UUGAGCGGCUCUGAACUUC[dT][dT] | |
| **RAG7-133** | | GCUCUUUGUCCGCUGAUCU[dT][dT] | | AGAUCAGCGGACAAAGAGC[dT][dT] | |
| **RAG7-162** | | UUCUUAGGGACUUGUUUUC[dT][dT] | | GAAAACAAGUCCCUAAGAA[dT][dT] | |
| **RAG7-694** | | AGGUCCUAUGCAUCCUCAU[dT][dT] | | AUGAGGAUGCAUAGGACCU[dT][dT] | |
| **RAG7-892** | | UGUUGGACCAGAAGUAAAG[dT][dT] | | CUUUACUUCUGGUCCAACA[dT][dT] | |
| **RAG7-177** | | AUUUGCCUUUGACCUUUCU[dT][dT] | | AGAAAGGUCAAAGGCAAAU[dT][dT] | |
| **RAG7-132** | | CUCUUUGUCCGCUGAUCUC[dT][dT] | | GAGAUCAGCGGACAAAGAG[dT][dT] | |
| **RAG7-178** | | AAUUUGCCUUUGACCUUUC[dT][dT] | | GAAAGGUCAAAGGCAAAUU[dT][dT] | |
| **RAG7-846** | | AAGGUUCCGAGGGGCCAUU[dT][dT] | | AAUGGCCCCUCGGAACCUU[dT][dT] | |
| **RAG7-139** | | UUUCCUGCUCUUUGUCCGC[dT][dT] | | GCGGACAAAGAGCAGGAAA[dT][dT] | |
| **RAG7-707** | | UUCUUCUCAGCCCAGGUCC[dT][dT] | | GGACCUGGGCUGAGAAGAA[dT][dT] | |
