# Supplementary figures and images for: Upregulation of LHPP by saRNA inhibited hepatocellular cancer cell proliferation and xenograft tumor growth
Source: PLoS One. 2024 May 2;19(5):e0299522. doi: 10.1371/journal.pone.0299522 (PMC11065268; doi:10.1371/journal.pone.0299522)

Marker Lane

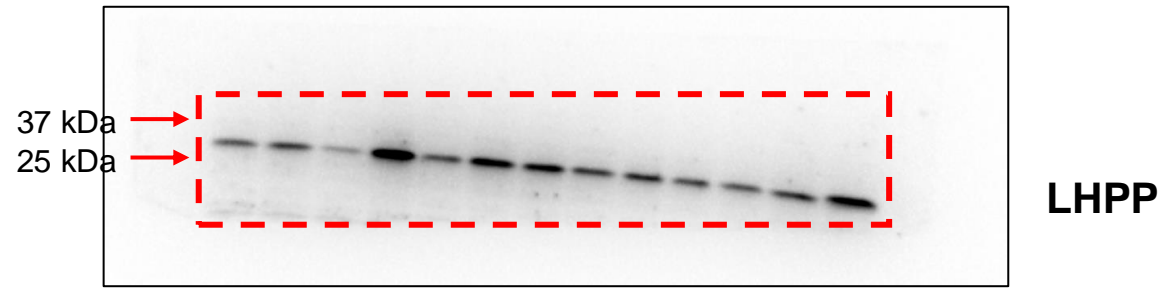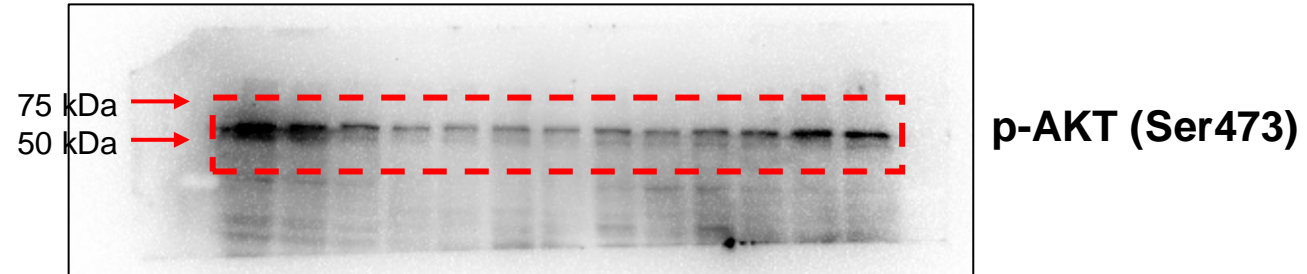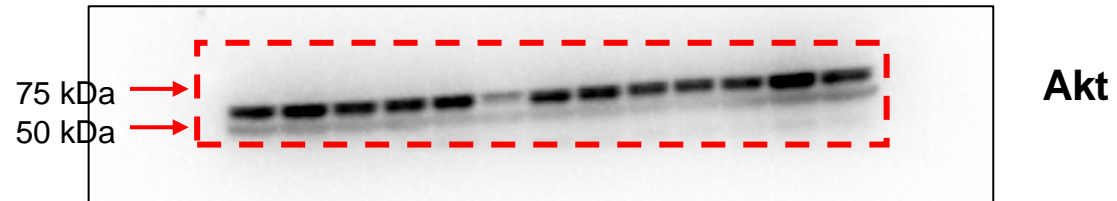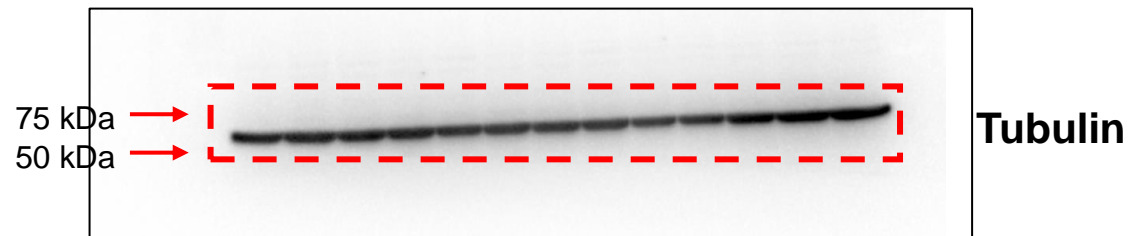

Mock dsCon2 siLHPP1 RAG7-133 RAG7-162 RAG7-694 RAG7-892 RAG7-177 RAG7-132 RAG7-178 RAG7-846 RAG7-139 RAG7-707

Detection: ECL

Supplement: S1 Raw images — (PDF) [file pone.0299522.s002.pdf]
